# Primary tumour immune response and lymph node yields in colon cancer

**DOI:** 10.1038/s41416-022-01700-1

**Published:** 2022-01-18

**Authors:** Nikhil Lal, Dedrick Kok Hong Chan, Minn E Ng, Louis Vermeulen, Simon James Alexander Buczacki

**Affiliations:** 1 Nuffield Department of Surgical Sciences, Old Road Campus Research Building (ORCRB), University of Oxford, Oxford, UK; 2 Department of Oncology, Old Road Campus Research Building (ORCRB), University of Oxford, Oxford, UK; 3 Laboratory for Experimental Oncology and Radiobiology (LEXOR), Center for Experimental Molecular Medicine (CEMM), Amsterdam UMC, University of Amsterdam, Amsterdam, The Netherlands; 4 Oncode Institute, Amsterdam, The Netherlands; 5 Oxford Colorectal Unit, Churchill Hospital, Oxford University Hospitals NHS Trust, Old Road, Oxford, UK

**Keywords:** Surgical oncology, Colon cancer

## Abstract

**Background:**

The mechanism underlying improved survival in non-metastatic colon cancer with higher lymph node (LN) yield is unknown. This study aimed to identify whether molecular features in the primary tumour were predictive of LN yield.

**Methods:**

Clinical, genomic, transcriptomic, proteomic and methylation data of non-metastatic, colon cancers studied in The Cancer Genome Atlas were interrogated for associations with LN yield. Based on maximal survival effects, patients were segregated into high (>15) and low (≤15) LN yield. Gene set enrichment analysis was performed on transcriptomic changes to identify biological processes associated with LN yield. Correlations were validated in an independent set of Stage II colon cancers.

**Results:**

High LN yield was found predictive of overall and disease-free survival. There was no association of higher LN yield and increasing nodal positivity. High LN yield was strongly linked with gene expression changes associated with the adaptive and dendritic cell immune response. This association was most prominent in node-negative cancers. Analogous findings were reproduced in the validation dataset.

**Conclusion:**

The study shows a strong association of an activated immune response in tumours with a high LN yield. Immunogenic tumours have a better prognosis, likely explaining the survival benefit with higher LN yields.

## Introduction

Lymph node (LN) yield in colon cancer resections is considered crucial for adequate staging and guiding adjuvant treatment [1]. The number of the LNs sampled has also been demonstrated as an independent prognostic marker for improved survival in both node-positive and node-negative disease [2]. Previous studies have highlighted that LN yield is multifactorial being dependent on surgeon, pathologist and patient-related factors [3, 4]. Currently, the American Joint Commission on Cancer (AJCC) and the National Quality Forum recommend a minimum number of 12 LNs for the adequate staging of colorectal cancers (CRC) [5]. Recently more radical surgical techniques including complete mesocolic excision (CME) with central vessel ligation (CVL) have been developed that improve LN yields in colon cancer resections [6, 7].

The mechanism by which higher LN yields are associated with improved survival is unclear. Traditional Halstedian views propose that removal of involved LNs prevents further spread of the disease from these deposits [8]. This view, however, cannot directly explain the clear survival benefit of high LN counts seen in the node-negative disease. In the field of breast cancer surgery, the Cady–Fisher model advocates that LN involvement is a marker of systemic disease and metastasis is influenced by intrinsic tumour–host interactions [9]. The model, therefore, proposes lymphadenectomy for staging alone. Stage migration, also known as the Will Rogers phenomenon, may thus explain the survival benefit seen in node-negative disease with high LN yields [10]. In support, Derwinger et al. observed a trend in stage migration from Stage I/II to Stage III, along with significantly improved survival, in patients with a greater LN yield in CRC resections [11]. However, several studies have contested this theory [12–14]. Bui et al. noted that LN positivity rates did not increase with a greater LN yield after colon cancer resections [13]. In 2011, Parsons and colleagues built on this by reporting that node-negative disease and a high LN yield had a lower 5-year mortality compared to node-positive disease [14].

Recently, some groups have hypothesised that a greater LN yield occurs due to an enhanced immune response generated by the host against the tumour [15, 16]. Markl et al. proposed LN size as a surrogate marker of immune activation, showing that LN size correlated significantly with LN yield and was a prognostic marker in pT3/pT4 node-negative disease [17]. Similarly, other studies have highlighted that lymphocytic and inflammatory cell infiltration in the primary tumour positively impact survival and are associated with the number of nodes harvested [18, 19]. Whilst these studies are useful contributions, it remains unclear whether the weak correlations seen are primary or secondary effects partly because of the limited and pre-selected number of measured parameters.

The advent of large, highly annotated multiomic datasets provides a unique opportunity for unbiased bioinformatic analyses to identify tumour molecular correlates with LN yield. Here, we applied this approach to search for biological signatures associated with high LN yield. Using a discovery and validation dataset, we interrogated genomic, transcriptomic and proteomic data to identify innate primary tumour molecular features that are associated with enhanced LN yield in non-metastatic colon cancer.

## Methods

### Discovery set

The Cancer Genome Atlas (TCGA) for CRC adenocarcinoma (COAD-READ) dataset was used to extract the discovery set [20]. The study was filtered to include samples of non-metastatic colon cancers of Stage l–lll, as defined in the 8th edition of the AJCC [5]. Tumours receiving neoadjuvant treatment were excluded from the study due to the possible impact of neoadjuvant therapy on the LN yield [21]. Tumours without annotation on the number of LN were excluded. Data including clinical attributes, overall & disease-free survival, genomic alterations, mRNA expression, protein expression and DNA methylation were extracted from the selected cohort. The minimum LN yield relating to the greatest survival benefit were generated for different LN cut-offs with Kaplan–Meier (KM) estimates using the R software package version 4.0.3 [22]. CMS subtypes were determined using the CMScaller R package [23]. MSI tumours were defined as those having a MSIsensor score of >3.5.

### Statistical analysis

Data were analysed in R, cBioPortal and Prism v6 [22, 24–27]. Non-parametric data were summarised tested using the Wilcoxon test for paired groups, Chi-squared test or Fisher’s exact test for two independent groups and Kruskal Wallis test for three or more groups. Parametric data were summarised using the Student’s *t* test for two-group analyses. For three or more groups ANOVA was performed. Multiple testing was performed using Benjamini–Hochberg FDR correction.

Gene Set Enrichment Analysis (GSEA) was performed using the GSEA 4.1.0 software package [7]. ‘Hallmark’ gene sets (50 gene sets) were obtained from the molecular signatures database (MSigDB v4.0) [28]. Each ‘hallmark’ gene set summarises multiple founder sets relating to a specific biological process or state. To obtain a mechanistic overview of increased LN yield, ranked transcriptomic data were analysed against the ‘hallmark’ gene sets. Upregulated and downregulated gene sets relating to a high and low LN yield were obtained. Normalised enrichment score (NES) for each gene set was evaluated after adjustment for multiple hypothesis testing. Further subgroup analyses were performed by running the ranked transcriptomic gene lists against the ‘hallmark’ gene sets for node-positive and node-negative disease. A false discovery rate (FDR) of <0.05 was treated as a significant event for all the analyses. Ranked gene lists for transcriptomic data for all samples, node-negative and node-positive disease were tested separately against the estimated immune signatures [29] and xCell type signatures [30].

### Validation set

A highly annotated profile of 90 AJCC Stage ll colon cancers undergoing curative surgery between 1997 and 2006 at the Academic Medical Centre (AMC), The Netherlands, were included as the validation cohort (AMC-AJCCII-90) [31]. Similar to the discovery set, a ranked list of differentially expressed genes between high (>15) and low (≤15) LN yield samples were generated (GSE33113) using GEO2R and tested against the ‘hallmark’ gene sets, immune signatures and xCell type signatures for enrichment [32].

## Results

### High LN yield in colon cancer surgery is positively associated with survival

**Fig. 1:**
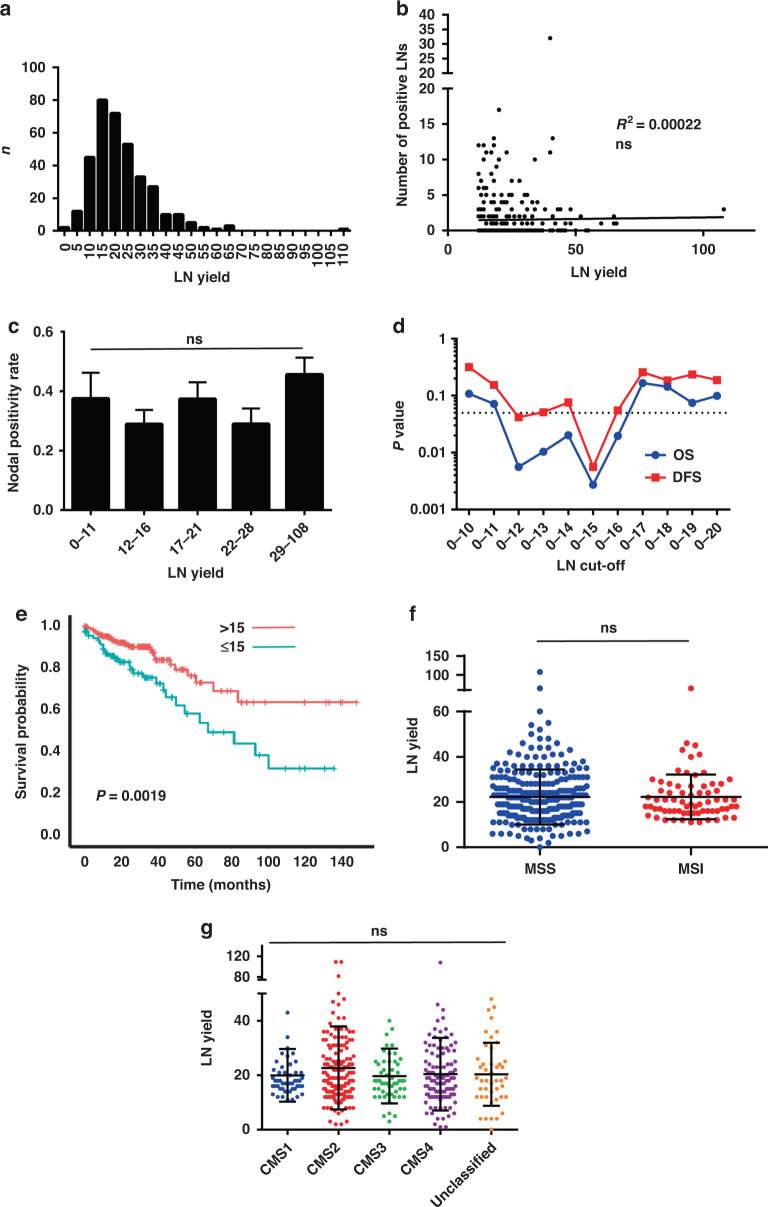
Stage migration fails to explain the survival benefit of high lymph node yield in TCGA colon cancer resections. **a** Histogram of LN yield distribution of TCGA-COAD tumours. **b** Scatter plot of the association of LN yield with the number of positive LNs found in the specimen. ns = not significant. **c** Histogram of the nodal positivity rate when compared to LN yield. ns = not significant. **d** Significance values for overall and disease-free survival differences for cut-off values of LN yields between 10 and 20. **e** Kaplan–Meier for the overall survival estimates using a cut-off of 15 LNs. **f** Column scatter plot comparing lymph node yields between microsatellite stable (MSS) and microsatellite unstable (MSI) colon tumours. ns = not significant. **g** Column scatter plot of LN yields for each consensus molecular subtype (CMS) of colon tumours. ns = not significant.

(TCGA) Colorectal (COAD) data were filtered to include all non-metastatic, chemotherapy naive, node-negative and node-positive colon cancer resections (*n* = 377) [20]. The median age at diagnosis was 69 (range: 31–90), and a larger proportion of patients were male (*n* = 200, 53.05%) (Supplementary Table 1). There were 230 right-sided tumours compared to 147 left-sided tumours. All patients were diagnosed with colon cancer during a 15-year period, ranging from 1998 to 2013. Moreover, 356 patients had information on the number of LN examined and the median number of LNs retrieved was 20 (range: 2–108) (Fig. 1a). In patients with an adequate LN assessment (≥12) there was no association with higher LN yield and the number of positive nodes identified (Fig. 1b). Similarly, there was no association found between higher LN yield and being nodal positive for all tumours (Fig. 1c). To define a clinically relevant LN cut-off that segregated patients, survival estimates were generated based on LN yield cut-offs between 10 and 20 (Fig. 1d). Compatible with previous reports it was found that segregating patients (node-negative and -positive) to ≤15 LN (*n* = 105) and >15 LN (*n* = 251) provided the most statistically significant difference in both overall and disease-specific survival (OS; *P*: 0.0019, 95% confidence interval (CI): 85.95–109.32 months, chi-square value: 9.575, hazard ratio: 0.459 (95% CI: 0.277– 0.76), DFS; *P*: 0.0037, 95% CI: 85.14–109.39 months, chi-square value: 8.394, hazard ratio: 1.445 (95% CI: 1.120–1.863)) (Fig. 1e). TCGA data were then mined to look for statistically significant clinical correlations with LN yield. Patient weight (*P* = 0.00007), height (*P* = 0.0281) and the absence of vascular invasion (*P* = 0.024) were found positively correlated with LN yield >15; however, on multiple testing only patient weight was found significant (*q* = 0.001) (Supplementary Table 2). Importantly, there was no association of LN yield with the presence of microsatellite instability (MSI) (Fig. 1f). Further, there was no association of LN yield with any of the consensus molecular subtypes (CMS) of colorectal cancer including hypermutated CMS1 and the unclassified group (Fig. 1g).

### Primary tumour immune response is associated with LN yield

**Fig. 2:**
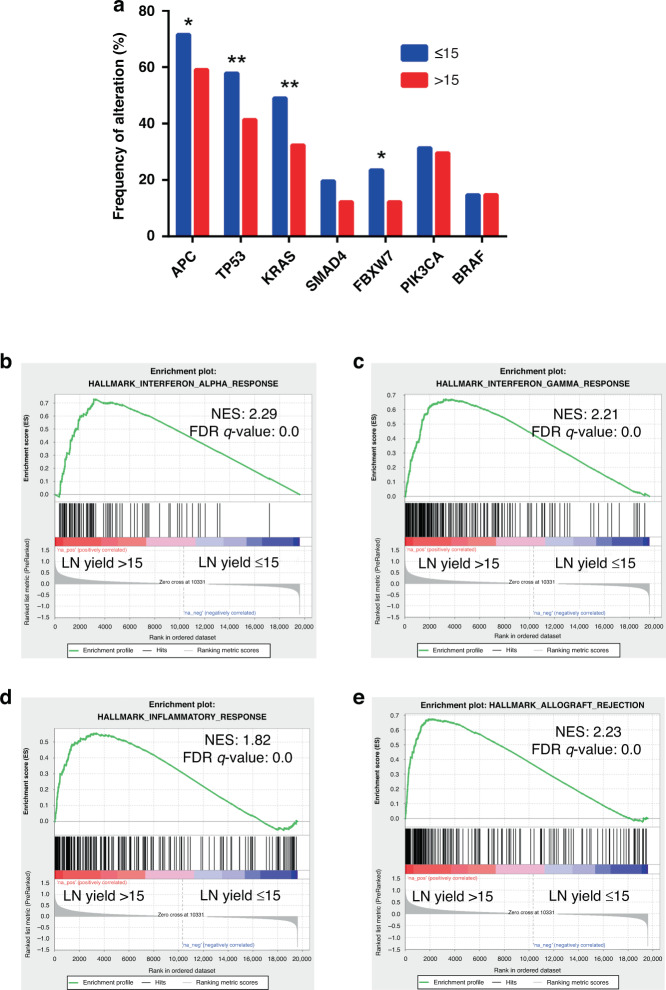
Hallmark geneset enrichments in high vs low LN yield colon cancers. **a** Histogram showing the frequency of the most common driver mutations found in tumours with high (>15) and low (≤15) LN yields. *P < 0.05, **P < 0.005. **b**–**e** GSEA plots demonstrating the degree of enrichment of the most significant hallmark signatures in high LN yield tumours.

Next, molecular changes (somatic mutations, copy number alterations, differentially expressed genes, protein levels and methylation changes) were analysed for associations with LN yield. Of the common CRC driver mutations, *APC* (*P* = 0.0188), *TP53* (*P* = 0.004), *KRAS* (*P* = 0.003) and *FBXW7* (*P* = 0.024) were found more frequently mutated in low LN yield patients but did not reach statistical significance at multiple testing (Fig. 2a). CLDN7, PRKCB, EEF2 and TFRC protein levels were all found at higher levels in high LN yield tumours (*q* = 0.0283). *VRK2* methylation levels were also found at higher levels in high LN yield patients (*q* = 0.006). To explore transcriptomic changes, GSEA was employed on a ranked gene list of differentially expressed genes between high and low LN yield cancers. Strikingly, a highly significant enrichment for ‘hallmark’ gene sets associated with immune response in patients with high LN yield was found including INTERFERON_ALPHA_RESPONSE (*q* < 0.005), INTERFERON_GAMMA_RESPONSE (*q* < 0.005), ALLOGRAFT_REJECTION (*q* < 0.005), and INFLAMMATORY_REPONSE (*q* < 0.005) (Fig. 2b–e and Supplementary Table 3). Gene sets associated with a low LN yield were dominated by processes associated with cellular proliferation including E2F_TARGETS (*q* < 0.005), MYC_TARGETS (*q* < 0.005) and the G2M_CHECKPOINT (*q* < 0.005) (Supplementary Fig. 1). These data suggest that LN yield may be driven by an enhanced immune response in the primary tumour making nodes easier to identify ex vivo rather than intrinsic surgical or pathologist-specific factors.

### The tumour immune response in high LN yield tumours is attenuated when there is nodal involvement

**Fig. 3:**
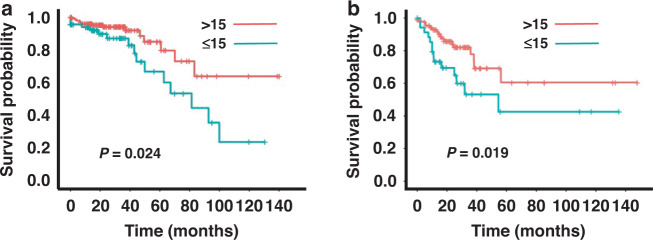
Kaplan–Meier overall survival (OS) estimates using a cut-off of 15 LNs. **a** OS for lymph node-negative tumours. **b** OS for lymph node-positive tumours. Node-negative; OS; p: 0.024, 95% confidence interval (CI): 81.73–109.18 months, chi-square value: 5.079, hazard ratio: 2.25 (95% CI: 1.09–4.65). Node-positive; OS; p: 0.019, 95% CI: 75.55–111.21 months, chi-square value: 5.478, hazard ratio: 2.276 (95% CI: 1.121–4.624).

To further explore these observations, tumours were segregated into node-negative and node-positive groups. KM estimates were generated using a 15 LN cut-off for both groups and an overall survival benefit of high LN yield was found in both (Fig. 3a, b). To ascertain whether there were biological differences in high and low LN yield between node-negative and positive groups, molecular data from both groups were analysed, using GSEA ‘hallmark’ gene sets for transcriptomic analysis as previous. There were no genetic alterations, methylation changes or protein expression differences that reached statistical significance for high or low LN yield in both node-negative and node-positive tumours. GSEA however demonstrated very similar findings to the earlier grouped analysis with both LN positive and LN negative tumours showing highly significant associations of immune response with high LN yield (Supplementary Tables 4 and 5). However, it was noted that in all cases the enrichment scores for immune-related gene sets were consistently weaker in node-positive tumours compared to node-negative (Table 1). These findings suggest that the primary tumour immune response that drives high LN yield becomes less effective when tumours have progressed, and LN involvement is present.

**Table 1:** Table of the enrichment scores of immune-related gene sets between node-negative and node-positive high LN yield (>15) tumours.

	Node negative	Node positive
INTERFERON_ALPHA	0.69	0.64
INTERFERON_GAMMA	0.66	0.5
INFLAMMATORY_RESPONSE	0.59	0.38
IL2_STAT5	0.55	0.33
IL6_JAK_STAT3	0.6	0.29

### High LN yield node-negative tumours have an enhanced adaptive immune response

**Fig. 4:**
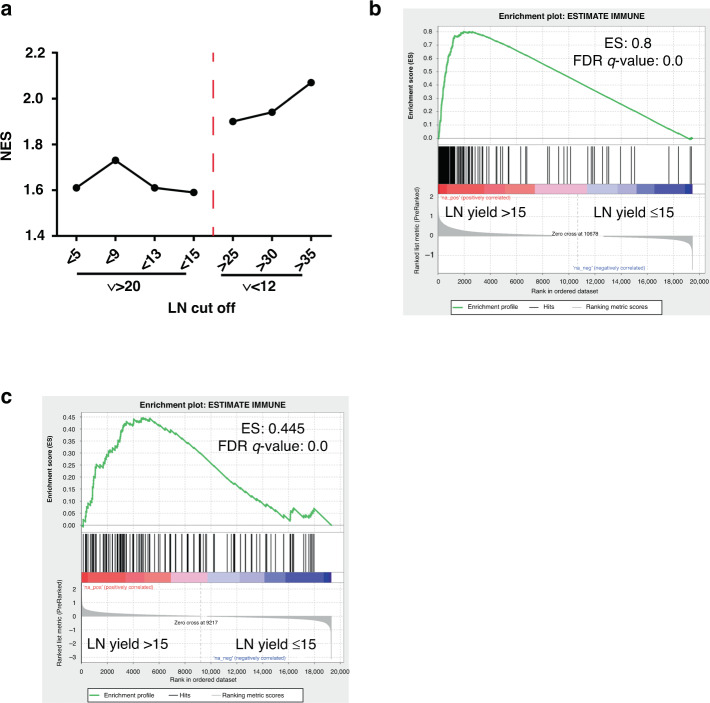
Relative immune response in node-negative and node-positive colon cancers. **a** Linked column chart of the inflammation gene set normalised enrichment scores (NES) for different cut-offs of LNs in node-negative colon cancers. **b** GSEA plot for the Estimate immune signature in node-negative colon cancers ranked by differential expression between high and low LN yield cancers. **c** GSEA plot for the Estimate immune signature in node-positive colon cancer ranked by differential expression between high and low LN yield cancers.

Theoretically, the association of LN yield with immune response found using GSEA could be driven by two possibilities: high LN yield tumours could have an elevated immune response or low LN yield tumours may have an inadequate response. To explore these options, differentially expressed gene lists were generated for different groups of node-negative tumours. First, the high number of LNs was fixed at ≥20 and the lower cut-off set at ≤15, ≤13, ≤9 and ≤5. The inflammatory response ‘hallmark’ gene set showed no change in enrichment scores using GSEA with lower cut-offs suggesting that the immune response in low LN yield tumours does not vary (Fig. 4a). Conversely, when fixing the lower cut-off at ≤12 and changing the higher cut-off to ≥25, ≥30 and ≥35 a clear trend of increasing enrichment of the inflammation gene set was observed (Fig. 4a). To further explore this all ‘hallmark’ gene sets were tested against the variable high cut-off tumours. Predictably there was a large overlap of shared gene sets enriched in these high LN groups (30/41). Of these 30 gene sets, 7 showed a significant positive correlation with increasing LN yield: KRAS_SIGNALLING_UP, NOTCH_SIGNALLING, UV_RESPONSE_UP, TGF_BETA_SIGNALLING, INFLAMMATORY_RESPONSE, IL2_STAT5_SIGNALLING and ANDROGEN_RESPONSE (Supplementary Table 6).

The Estimate immune signature and xCell cell-type signatures were next used to independently confirm the apparent high immune response in tumours with high LN yield and explore whether specific subsets of immune cells were enriched in these tumours [29, 30]. GSEA using the Estimate signature validated the stronger enrichment of immune-related genes in node-negative high LN yield tumours compared to node-positive (Fig. 4b, c). Analysis of the xCell gene sets showed highly significant enrichment of signatures associated with B cells and T cells (CD4 + and CD8 + ) in node-negative tumours with a high LN yield patient suggesting a broad activation of the adaptive immune response in these tumours (Supplementary Table 7). Interestingly, a strong enrichment in dendritic cell signatures was also found. There was a statistically significant enrichment in some gene sets associated with the innate immune response (eosinophils, macrophages, mast cells, basophils, natural killer cells and neutrophils) but this was less strong than the adaptive enrichment.

### Validation set

An independent validation dataset was used to confirm the findings demonstrated using the TCGA data. As Stage ll tumours made the largest proportion of our discovery cohort and node-negative tumours demonstrated greater immunogenicity compared to node-positive, an AMC-AJCCll-90 cohort of a highly annotated transcriptomic dataset of node-negative colon cancers was used as the validation set [31]. Overall, the median age at the time of the operation was 73.4 (range: 34.6–95.1) and there were slightly more female patients than male (*n* = 42, 46.7%) (Supplementary Table 8). A larger proportion of cancers were right-sided (*n* = 52) compared to left-sided (*n* = 38). Of the 90 patients in the group, 84 had associated LN yield numbers. The median number of LNs reviewed were 12 (range: 1–46). Using a cut-off of 15 LNs, the cohort was segregated into low yield (*n* = 56) and high yield (*n* = 28) and a ranked differentially expressed gene list generated. GSEA using the ‘hallmarks’ gene sets demonstrated highly similar findings to that with the TCGA data and strong enrichment of gene sets associated with the immune response (Supplementary Table 9).

## Discussion

Conflicting data around Cady–Fisher or Halstedian explanations lends credence to an alternative explanation for the survival advantage associated with increased LN yield in colon cancer resections. Here, we approached this important clinical question from an unbiased bioinformatic perspective with the hypothesis that innate biological processes in the primary tumour underlie varying LN yields. Our approach, using primarily TCGA data, makes use of the largest most highly annotated and well-validated multiomic dataset currently available [20]. Furthermore, the contributions in the TCGA data are multi-institutional, thereby, including centres with varying surgical and pathological techniques to counter-selection bias. Our study reproduces the findings of others showing a clear survival benefit with increased LN yield in both node-negative and node-positive colon cancers [14, 15]. We also find that there is no association between higher LN yields and the number of positive nodes found nor the chance of being node-positive per se. Cumulatively, these findings argue against Halsted, Cady–Fisher and stage-migration mechanisms being an explanation for the profound survival effect seen.

**Fig. 5:**
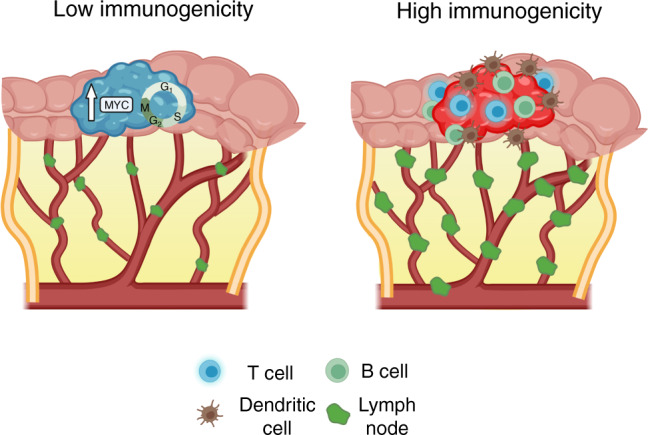
Schematic demonstrating the association between tumour immune status and lymph node yield in colorectal cancer.

Strikingly, our bioinformatic analysis shows a clear molecular association of enhanced immune response with increasing numbers of LNs retrieved in both node-negative and node-positive tumours. We hypothesise that a strong immune response in the primary tumour will generate more clearly visible nodes to the pathologist assessing the specimen and thus a higher yield (Fig. 5). Further, this strong immune response against the tumour will result in improved patient survival, as has been shown in many clinical studies [17, 18, 33]. The effect of local immune response on nodal hyperplasia resulting in enlarged lymph nodes has been studied previously [34]. We are not the first to find an association between immune response in the primary tumour and LN yield, however, the two previous studies we cite showed only weak associations and only in T cells [17, 18]. Kim et al. analysed T-cell makers (CD3, CD8 and CD45RO) finding positive associations with CD3 and CD8 levels and LN yield in Stage II/III colon and rectal resections [18]. Markl et al analysed CD3 and CD8 levels in Stage II colon cancer resections and found a positive association with CD3 levels only [17]. Our unbiased multiomic approach finds a broad B- and T-cell adaptive immune response is the dominant differentially regulated process between high and low LN yield Stage II and III colon cancer resections. We also describe, for the first time, a strong association between the presence of dendritic cells and high LN yields. This observation is biologically plausible given the well-described role of these cells in transporting cancer-associated antigens to draining LNs for T-cell priming and activation [35]. We find however that this broad immune response becomes attenuated in node-positive cancers. Biologically, this would also be predicted; as a tumour progresses the immune response may act to retard growth and nodal spread. Tumours that subsequently develop nodal involvement would perhaps not unsurprisingly have lost some of that local immune control.

It is interesting that we were unable to identify a clear underlying genetic or epigenetic process driving the apparent enhanced immune response in high LN yield colon cancers. Previous studies have suggested that MSI tumours have an increased LN yield although other groups have not replicated this [36, 37]. MSI tumours are believed to express high levels of neoantigens driving an enhanced immune response and account for their known susceptibility to checkpoint blockade [38]. In our analysis, we did not find MSI tumours to have higher LN yields, nor any of the recently defined CMS subtypes. These findings are similar to recent studies evaluating the impact of microsatellite instability on LN yield [39]. The contrasting evidence in the literature suggests a need to further study the immunogenicity of MSI tumours. Whilst *APC, TP53, KRAS* and *FBXW7* mutations were more common in low LN yield tumours this was not significant when multiple testing was applied. However, the association of MYC activation and an immunosuppressive environment as seen in low lymph node yield tumours has previously been described in other solid organ malignancies [40]. These findings will provide an important focus for further experimental study in colorectal cancer.

The most exciting recent development in colon cancer surgery has been the development of CME with CVL [6]. Oncological outcome data from the Erlangen group and others are persuasive although have been contested by others [41] and it is uncertain how this radical surgery achieves the apparent benefit [6]. Our study would suggest that the survival effects of these surgeries may not be driven by enhanced lymphadenectomy and perhaps rather the exquisite attention to planal surgery and preservation of an intact mesenteric peritoneum, although this hypothesis also remains to be tested. The increasing use of neoadjuvant chemotherapy also has the potential to confound the use of LN yield alone as a prognostic indicator [42]. These therapies have the potential to both perturb the potent antitumour effect of the immune system and may also obscure LN yield estimate postoperatively. Further studies will be required to better understand whether this is a valid concern.

In summary, we show that LN yield in colon cancer resections is determined by a host–tumour adaptive immune response and efforts to maximise LN yield in a contemporary era of pre-existing high-quality surgery and pathology may fail to further improve oncological outcomes.

## Supplementary information

Supplementary Legends

Supplementary Table and Figures

## Data Availability

Data are available within the article or its Supplementary Materials.
