# Supplementary material for: Primary tumour immune response and lymph node yields in colon cancer
Source: Br J Cancer. 2022 Jan 18;126(8):1178–85. doi: 10.1038/s41416-022-01700-1 (PMC9023574; doi:10.1038/s41416-022-01700-1)
Supplement: Supplementary file 1 — Supplementary Legends [file 41416_2022_1700_MOESM1_ESM.docx]

**Supplementary Legends**

**Supplementary Figure 1.** (A-C) GSEA plots of the most enriched hallmark gene sets in low LN yield colon tumours.

**Supplementary Table 1.**Demographics of patients included in the discovery cohort.

**Supplementary Table 2.**Clinical correlates with lymph node yield for all non-metastatic TCGA colon cancers

**Supplementary Table 3.**GSEA data of hallmark gene sets enriched or de-enriched in high LN yield non-metastatic TCGA cancers.

**Supplementary Table 4.**GSEA data of hallmark gene sets enriched or de-enriched in high LN yield non-metastatic node positive TCGA cancers.

**Supplementary Table 5.**GSEA data of hallmark gene sets enriched or de-enriched in high LN yield non-metastatic node negative TCGA cancers.

**Supplementary Table 6.**Regression analysis of trends in shared enriched GSEA gene sets with varying cut offs of node negative high LN yield (≤12 and changing the higher cut-off to ≥20, ≥25, ≥30 and ≥35).

**Supplementary Table 7.** GSEA of enriched and de-enriched xCell cell type signatures in node negative TCGA non-metastatic colon cancers

**Supplementary Table 8.**Demographics of patients in the validation set (AMC-AJCCll-90).

**Supplementary Table 9.**GSEA data of hallmark gene sets enriched or de-enriched in AMC-AJCCll-90.
