# Supplementary material for: Primary tumour immune response and lymph node yields in colon cancer
Source: Br J Cancer. 2022 Jan 18;126(8):1178–85. doi: 10.1038/s41416-022-01700-1 (PMC9023574; doi:10.1038/s41416-022-01700-1)

**Supplementary Table 1.**Demographics of patients included in the discovery cohort.

| Characteristics | 377 patients Stage I-III |
| --- | --- |
| Male sex (%) | 200(53.05%) |
| Median at age at the time of diagnosis  (range) | 69(31-90) |
| T-stage  -Tis  -T1  -T2  -T3  -T4 | 1(0.2%)  9(2.3%)  74(19.62%)  263(69.76%)  30(7.95%) |
| Stage  I  II  III | 74(19.63%)  177(46.69%)  127(33.69%) |
| Location  -Left  -Right | 230(61%)  147(39%) |
| Lymph node yield  (range) | 20(2-108) |
| Microsatellite Instability – High  Microsatellite Instability – Low | 70  266 |

**Supplementary Table 2.**Clinical correlates with lymph node yield for all non-metastatic TCGA colon cancers.

**Supplementary Table 3.**GSEA data of hallmark gene sets enriched or de-enriched in high LN yield non-metastatic TCGA cancers.

**Supplementary Table 4.**GSEA data of hallmark gene sets enriched or de-enriched in high LN yield non-metastatic node positive TCGA cancers.

**Supplementary Table 5.**GSEA data of hallmark gene sets enriched or de-enriched in high LN yield non-metastatic node negative TCGA cancers.

**Supplementary Table 6.** Regression analysis of trends in shared enriched GSEA gene sets with varying cut offs of node negative high LN yield (≤12 and changing the higher cut-off to ≥20, ≥25, ≥30 and ≥35).

**Supplementary Table 7.**GSEA of enriched and de-enriched xCell cell type signatures in node negative TCGA non-metastatic colon cancers

| **Cell Signature vs LN Neg - Upregulated genes** | |  |  |  |  |
| --- | --- | --- | --- | --- | --- |
| **NAME** | **GS<br> follow link to MSigDB** | **ES** | **NES** | **NOM p-val** | **FDR q-val** |
| B-CELLS_NOVERSHTERN_2 | B-CELLS_NOVERSHTERN_2 | 0.81829786 | 2.3930423 | 0 | 0 |
| B-CELLS_NOVERSHTERN_1 | B-CELLS_NOVERSHTERN_1 | 0.82927537 | 2.366363 | 0 | 0 |
| B-CELLS_NOVERSHTERN_3 | B-CELLS_NOVERSHTERN_3 | 0.841981 | 2.3470483 | 0 | 0 |
| CD4+ T-CELLS_HPCA_2 | CD4+ T-CELLS_HPCA_2 | 0.90191203 | 2.3168342 | 0 | 0 |
| CD4+ T-CELLS_HPCA_3 | CD4+ T-CELLS_HPCA_3 | 0.85698843 | 2.2893848 | 0 | 0 |
| CD8+ TEM_BLUEPRINT_3 | CD8+ TEM_BLUEPRINT_3 | 0.7895638 | 2.271392 | 0 | 0 |
| NAIVE B-CELLS_HPCA_3 | NAIVE B-CELLS_HPCA_3 | 0.8501962 | 2.267175 | 0 | 0 |
| MEMORY B-CELLS_HPCA_2 | MEMORY B-CELLS_HPCA_2 | 0.83918023 | 2.2514207 | 0 | 0 |
| CD4+ T-CELLS_HPCA_1 | CD4+ T-CELLS_HPCA_1 | 0.84301245 | 2.2400339 | 0 | 0 |
| B-CELLS_HPCA_1 | B-CELLS_HPCA_1 | 0.8218723 | 2.2265859 | 0 | 0 |
| CD4+ NAIVE T-CELLS_NOVERSHTERN_1 | CD4+ NAIVE T-CELLS_NOVERSHTERN_1 | 0.83511037 | 2.2197032 | 0 | 0 |
| MEMORY B-CELLS_IRIS_2 | MEMORY B-CELLS_IRIS_2 | 0.9279473 | 2.2100666 | 0 | 0 |
| MEMORY B-CELLS_BLUEPRINT_3 | MEMORY B-CELLS_BLUEPRINT_3 | 0.7618965 | 2.207972 | 0 | 0 |
| NAIVE B-CELLS_HPCA_2 | NAIVE B-CELLS_HPCA_2 | 0.8564154 | 2.197895 | 0 | 0 |
| MPP_FANTOM_2 | MPP_FANTOM_2 | 0.7751936 | 2.1910489 | 0 | 0 |
| MPP_FANTOM_1 | MPP_FANTOM_1 | 0.7786832 | 2.1776633 | 0 | 0 |
| NAIVE B-CELLS_HPCA_1 | NAIVE B-CELLS_HPCA_1 | 0.8635369 | 2.1775854 | 0 | 0 |
| MEMORY B-CELLS_BLUEPRINT_2 | MEMORY B-CELLS_BLUEPRINT_2 | 0.759176 | 2.1773937 | 0 | 0 |
| CD4+ TEM_NOVERSHTERN_1 | CD4+ TEM_NOVERSHTERN_1 | 0.8008186 | 2.173287 | 0 | 0 |
| DC_IRIS_2 | DC_IRIS_2 | 0.8353893 | 2.1703784 | 0 | 0 |
| CD8+ TEM_HPCA_2 | CD8+ TEM_HPCA_2 | 0.6904223 | 2.1663053 | 0 | 0 |
| CD4+ MEMORY T-CELLS_IRIS_2 | CD4+ MEMORY T-CELLS_IRIS_2 | 0.946153 | 2.1629076 | 0 | 0 |
| B-CELLS_FANTOM_3 | B-CELLS_FANTOM_3 | 0.89082795 | 2.1543374 | 0 | 0 |
| CD8+ TCM_HPCA_3 | CD8+ TCM_HPCA_3 | 0.7645629 | 2.1490073 | 0 | 0 |
| CD4+ TEM_HPCA_2 | CD4+ TEM_HPCA_2 | 0.7724087 | 2.1429229 | 0 | 0 |
| CD8+ TCM_NOVERSHTERN_1 | CD8+ TCM_NOVERSHTERN_1 | 0.8368682 | 2.1375103 | 0 | 0 |
| CD4+ NAIVE T-CELLS_IRIS_3 | CD4+ NAIVE T-CELLS_IRIS_3 | 0.7708857 | 2.1286714 | 0 | 0 |
| CD4+ MEMORY T-CELLS_IRIS_3 | CD4+ MEMORY T-CELLS_IRIS_3 | 0.875742 | 2.123479 | 0 | 0 |
| MEMORY B-CELLS_HPCA_1 | MEMORY B-CELLS_HPCA_1 | 0.9188361 | 2.1189134 | 0 | 0 |
| CD8+ TCM_NOVERSHTERN_2 | CD8+ TCM_NOVERSHTERN_2 | 0.8443677 | 2.1014214 | 0 | 0 |
| CD8+ TCM_BLUEPRINT_2 | CD8+ TCM_BLUEPRINT_2 | 0.7696813 | 2.0998027 | 0 | 0 |
| CD4+ TCM_HPCA_2 | CD4+ TCM_HPCA_2 | 0.7155705 | 2.09891 | 0 | 0 |
| CD4+ T-CELLS_BLUEPRINT_2 | CD4+ T-CELLS_BLUEPRINT_2 | 0.7295105 | 2.0983937 | 0 | 0 |
| CD4+ T-CELLS_BLUEPRINT_3 | CD4+ T-CELLS_BLUEPRINT_3 | 0.8299221 | 2.0933757 | 0 | 0 |
| CD4+ TCM_NOVERSHTERN_3 | CD4+ TCM_NOVERSHTERN_3 | 0.7371088 | 2.093126 | 0 | 0 |
| CD4+ T-CELLS_BLUEPRINT_1 | CD4+ T-CELLS_BLUEPRINT_1 | 0.79359126 | 2.0927877 | 0 | 0 |
| CD8+ TEM_NOVERSHTERN_3 | CD8+ TEM_NOVERSHTERN_3 | 0.8537032 | 2.0913453 | 0 | 0 |
| MPP_FANTOM_3 | MPP_FANTOM_3 | 0.81460017 | 2.084715 | 0 | 0 |
| CD8+ TEM_NOVERSHTERN_1 | CD8+ TEM_NOVERSHTERN_1 | 0.83783793 | 2.0801485 | 0 | 0 |
| B-CELLS_FANTOM_1 | B-CELLS_FANTOM_1 | 0.92281 | 2.073964 | 0 | 0 |
| NAIVE B-CELLS_BLUEPRINT_2 | NAIVE B-CELLS_BLUEPRINT_2 | 0.8893086 | 2.0675921 | 0 | 0 |
| MONOCYTES_NOVERSHTERN_1 | MONOCYTES_NOVERSHTERN_1 | 0.6957637 | 2.0673685 | 0 | 0 |
| DC_IRIS_3 | DC_IRIS_3 | 0.8820074 | 2.0435772 | 0 | 0 |
| CD8+ T-CELLS_HPCA_3 | CD8+ T-CELLS_HPCA_3 | 0.8191196 | 2.0422204 | 0 | 0 |
| CD4+ TCM_HPCA_3 | CD4+ TCM_HPCA_3 | 0.7761989 | 2.0407462 | 0 | 0 |
| CD8+ TEM_NOVERSHTERN_2 | CD8+ TEM_NOVERSHTERN_2 | 0.85931224 | 2.040667 | 0 | 0 |
| CD4+ NAIVE T-CELLS_FANTOM_2 | CD4+ NAIVE T-CELLS_FANTOM_2 | 0.7286708 | 2.0389817 | 0 | 0 |
| CDC_NOVERSHTERN_3 | CDC_NOVERSHTERN_3 | 0.8674305 | 2.0381243 | 0 | 0 |
| CLASS-SWITCHED MEMORY B-CELLS_NOVERSHTERN_1 | CLASS-SWITCHED MEMORY B-CELLS_NOVERSHTERN_1 | 0.8856738 | 2.037394 | 0 | 0 |
| MEMORY B-CELLS_BLUEPRINT_1 | MEMORY B-CELLS_BLUEPRINT_1 | 0.6552073 | 2.0328155 | 0 | 0 |
| TGD CELLS_HPCA_3 | TGD CELLS_HPCA_3 | 0.65644646 | 2.0298061 | 0 | 0 |
| TGD CELLS_HPCA_1 | TGD CELLS_HPCA_1 | 0.64922684 | 2.0173779 | 0 | 0 |
| CD8+ TCM_BLUEPRINT_3 | CD8+ TCM_BLUEPRINT_3 | 0.8770758 | 2.0057604 | 0 | 0 |
| TREGS_FANTOM_3 | TREGS_FANTOM_3 | 0.83798736 | 2.005571 | 0 | 0 |
| CD8+ TEM_BLUEPRINT_2 | CD8+ TEM_BLUEPRINT_2 | 0.64890814 | 2.0013094 | 0 | 0 |
| DC_FANTOM_3 | DC_FANTOM_3 | 0.83925873 | 1.998847 | 0 | 0 |
| CD4+ TCM_HPCA_1 | CD4+ TCM_HPCA_1 | 0.70252234 | 1.9875869 | 0 | 0 |
| NK CELLS_BLUEPRINT_3 | NK CELLS_BLUEPRINT_3 | 0.7892877 | 1.9733181 | 0 | 0 |
| CD8+ TCM_HPCA_1 | CD8+ TCM_HPCA_1 | 0.7948756 | 1.9725413 | 0 | 0 |
| CD8+ TEM_HPCA_1 | CD8+ TEM_HPCA_1 | 0.63228214 | 1.969265 | 0 | 0 |
| EOSINOPHILS_FANTOM_3 | EOSINOPHILS_FANTOM_3 | 0.7325983 | 1.9650837 | 0 | 0 |
| TGD CELLS_HPCA_2 | TGD CELLS_HPCA_2 | 0.62862074 | 1.9560425 | 0 | 1.51E-05 |
| EOSINOPHILS_FANTOM_1 | EOSINOPHILS_FANTOM_1 | 0.7369889 | 1.954733 | 0 | 1.49E-05 |
| EOSINOPHILS_FANTOM_2 | EOSINOPHILS_FANTOM_2 | 0.74092776 | 1.9405928 | 0 | 5.95E-05 |
| CD8+ T-CELLS_BLUEPRINT_3 | CD8+ T-CELLS_BLUEPRINT_3 | 0.8038276 | 1.930323 | 0 | 5.86E-05 |
| PLASMA CELLS_HPCA_2 | PLASMA CELLS_HPCA_2 | 0.72911423 | 1.9289463 | 0 | 5.77E-05 |
| CD4+ TCM_NOVERSHTERN_1 | CD4+ TCM_NOVERSHTERN_1 | 0.78688455 | 1.924313 | 0 | 7.05E-05 |
| NK CELLS_FANTOM_1 | NK CELLS_FANTOM_1 | 0.8432415 | 1.9131747 | 0 | 6.95E-05 |
| CD4+ NAIVE T-CELLS_NOVERSHTERN_3 | CD4+ NAIVE T-CELLS_NOVERSHTERN_3 | 0.83139575 | 1.898969 | 0 | 1.24E-04 |
| CD4+ TCM_NOVERSHTERN_2 | CD4+ TCM_NOVERSHTERN_2 | 0.8045649 | 1.8923633 | 0 | 1.22E-04 |
| CD8+ TEM_HPCA_3 | CD8+ TEM_HPCA_3 | 0.69380814 | 1.8879598 | 0 | 1.20E-04 |
| ADC_IRIS_2 | ADC_IRIS_2 | 0.6092411 | 1.8799464 | 0 | 1.45E-04 |
| PLASMA CELLS_IRIS_3 | PLASMA CELLS_IRIS_3 | 0.7913056 | 1.8797297 | 0 | 1.43E-04 |
| NAIVE B-CELLS_NOVERSHTERN_3 | NAIVE B-CELLS_NOVERSHTERN_3 | 0.65080625 | 1.8785083 | 0 | 1.41E-04 |
| PLASMA CELLS_HPCA_3 | PLASMA CELLS_HPCA_3 | 0.8161302 | 1.8771579 | 0 | 1.39E-04 |
| CD4+ TEM_BLUEPRINT_3 | CD4+ TEM_BLUEPRINT_3 | 0.7757249 | 1.8759662 | 0 | 1.38E-04 |
| PLASMA CELLS_HPCA_1 | PLASMA CELLS_HPCA_1 | 0.77620375 | 1.8758726 | 0 | 1.36E-04 |
| CD8+ T-CELLS_NOVERSHTERN_3 | CD8+ T-CELLS_NOVERSHTERN_3 | 0.6918086 | 1.8731884 | 0 | 1.34E-04 |
| CD4+ T-CELLS_FANTOM_3 | CD4+ T-CELLS_FANTOM_3 | 0.6429724 | 1.8729402 | 0 | 1.32E-04 |
| CD4+ NAIVE T-CELLS_FANTOM_1 | CD4+ NAIVE T-CELLS_FANTOM_1 | 0.83139575 | 1.8729026 | 0 | 1.31E-04 |
| PDC_FANTOM_3 | PDC_FANTOM_3 | 0.7519547 | 1.8718287 | 0 | 1.29E-04 |
| DC_FANTOM_2 | DC_FANTOM_2 | 0.65105486 | 1.8707985 | 0 | 1.28E-04 |
| CD8+ T-CELLS_NOVERSHTERN_1 | CD8+ T-CELLS_NOVERSHTERN_1 | 0.721727 | 1.8608612 | 0 | 1.83E-04 |
| HEPATOCYTES_FANTOM_2 | HEPATOCYTES_FANTOM_2 | 0.60094494 | 1.8581004 | 0 | 1.92E-04 |
| MACROPHAGES_BLUEPRINT_1 | MACROPHAGES_BLUEPRINT_1 | 0.63684684 | 1.842035 | 0 | 2.34E-04 |
| NAIVE B-CELLS_NOVERSHTERN_2 | NAIVE B-CELLS_NOVERSHTERN_2 | 0.6935807 | 1.8417591 | 0 | 2.32E-04 |
| CD8+ T-CELLS_FANTOM_3 | CD8+ T-CELLS_FANTOM_3 | 0.6918086 | 1.8398669 | 0 | 2.62E-04 |
| CD8+ T-CELLS_FANTOM_1 | CD8+ T-CELLS_FANTOM_1 | 0.65407014 | 1.839726 | 0 | 2.59E-04 |
| ADC_IRIS_1 | ADC_IRIS_1 | 0.76512325 | 1.8384933 | 0 | 2.67E-04 |
| CD4+ NAIVE T-CELLS_IRIS_1 | CD4+ NAIVE T-CELLS_IRIS_1 | 0.7992603 | 1.8379743 | 0 | 2.64E-04 |
| TREGS_BLUEPRINT_1 | TREGS_BLUEPRINT_1 | 0.75147945 | 1.8373729 | 0.00128866 | 2.61E-04 |
| CD4+ T-CELLS_FANTOM_1 | CD4+ T-CELLS_FANTOM_1 | 0.7371224 | 1.8353786 | 0 | 2.58E-04 |
| EOSINOPHILS_NOVERSHTERN_1 | EOSINOPHILS_NOVERSHTERN_1 | 0.80213463 | 1.8339903 | 0.00137174 | 2.86E-04 |
| MONOCYTES_FANTOM_1 | MONOCYTES_FANTOM_1 | 0.61519915 | 1.8298154 | 0 | 3.14E-04 |
| EOSINOPHILS_NOVERSHTERN_3 | EOSINOPHILS_NOVERSHTERN_3 | 0.8078179 | 1.8159478 | 0 | 4.51E-04 |
| NK CELLS_BLUEPRINT_2 | NK CELLS_BLUEPRINT_2 | 0.6904406 | 1.8124245 | 0 | 4.86E-04 |
| MACROPHAGES M1_FANTOM_3 | MACROPHAGES M1_FANTOM_3 | 0.61386883 | 1.8066447 | 0 | 5.40E-04 |
| NK CELLS_HPCA_1 | NK CELLS_HPCA_1 | 0.64848995 | 1.8051486 | 0 | 5.54E-04 |
| NAIVE B-CELLS_NOVERSHTERN_1 | NAIVE B-CELLS_NOVERSHTERN_1 | 0.6558825 | 1.8010801 | 0 | 5.97E-04 |
| CD8+ T-CELLS_BLUEPRINT_2 | CD8+ T-CELLS_BLUEPRINT_2 | 0.65407014 | 1.8008976 | 0 | 5.91E-04 |
| CD8+ T-CELLS_BLUEPRINT_1 | CD8+ T-CELLS_BLUEPRINT_1 | 0.6935094 | 1.7942921 | 0.00122249 | 6.43E-04 |
| PLASMA CELLS_IRIS_2 | PLASMA CELLS_IRIS_2 | 0.67334366 | 1.7860268 | 0 | 7.12E-04 |
| CD8+ NAIVE T-CELLS_HPCA_3 | CD8+ NAIVE T-CELLS_HPCA_3 | 0.70365477 | 1.7856556 | 0.00126103 | 7.05E-04 |
| CLASS-SWITCHED MEMORY B-CELLS_BLUEPRINT_3 | CLASS-SWITCHED MEMORY B-CELLS_BLUEPRINT_3 | 0.73782843 | 1.7825131 | 0.00132626 | 7.26E-04 |
| NK CELLS_HPCA_2 | NK CELLS_HPCA_2 | 0.6711312 | 1.7809943 | 0.00121951 | 7.28E-04 |
| MONOCYTES_FANTOM_3 | MONOCYTES_FANTOM_3 | 0.57421595 | 1.7760985 | 0 | 7.67E-04 |
| EOSINOPHILS_BLUEPRINT_3 | EOSINOPHILS_BLUEPRINT_3 | 0.6421141 | 1.775766 | 0.00115607 | 7.60E-04 |
| HEPATOCYTES_FANTOM_1 | HEPATOCYTES_FANTOM_1 | 0.5665903 | 1.7685877 | 0 | 8.24E-04 |
| NK CELLS_IRIS_2 | NK CELLS_IRIS_2 | 0.737886 | 1.7656674 | 0 | 8.96E-04 |
| HEPATOCYTES_FANTOM_3 | HEPATOCYTES_FANTOM_3 | 0.56906545 | 1.763133 | 0 | 9.39E-04 |
| PRO B-CELLS_NOVERSHTERN_3 | PRO B-CELLS_NOVERSHTERN_3 | 0.7923714 | 1.7630879 | 0.00134771 | 9.31E-04 |
| MACROPHAGES M1_FANTOM_2 | MACROPHAGES M1_FANTOM_2 | 0.5933023 | 1.758208 | 0 | 0.00104197 |
| MONOCYTES_FANTOM_2 | MONOCYTES_FANTOM_2 | 0.5601495 | 1.7578207 | 0 | 0.00106672 |
| MAST CELLS_FANTOM_3 | MAST CELLS_FANTOM_3 | 0.60372066 | 1.756586 | 0 | 0.00107466 |
| NK CELLS_HPCA_3 | NK CELLS_HPCA_3 | 0.66548735 | 1.7557204 | 0.00118906 | 0.00107384 |
| MELANOCYTES_FANTOM_2 | MELANOCYTES_FANTOM_2 | 0.5704415 | 1.7529705 | 0 | 0.00110606 |
| MACROPHAGES M1_BLUEPRINT_3 | MACROPHAGES M1_BLUEPRINT_3 | 0.58322966 | 1.7523469 | 0 | 0.00110479 |
| MACROPHAGES_BLUEPRINT_3 | MACROPHAGES_BLUEPRINT_3 | 0.5972086 | 1.7439407 | 0 | 0.00125744 |
| ADC_IRIS_3 | ADC_IRIS_3 | 0.57913756 | 1.7414951 | 0 | 0.00128714 |
| BASOPHILS_NOVERSHTERN_1 | BASOPHILS_NOVERSHTERN_1 | 0.6381492 | 1.7291396 | 0.00235571 | 0.00160221 |
| MELANOCYTES_FANTOM_3 | MELANOCYTES_FANTOM_3 | 0.5441502 | 1.7207886 | 0 | 0.00180974 |
| EOSINOPHILS_BLUEPRINT_1 | EOSINOPHILS_BLUEPRINT_1 | 0.74618113 | 1.7162143 | 0.0052356 | 0.00197347 |
| CD8+ T-CELLS_HPCA_2 | CD8+ T-CELLS_HPCA_2 | 0.71532357 | 1.715035 | 0.00269542 | 0.00199632 |
| CLASS-SWITCHED MEMORY B-CELLS_BLUEPRINT_2 | CLASS-SWITCHED MEMORY B-CELLS_BLUEPRINT_2 | 0.6784921 | 1.7004482 | 0.00246914 | 0.0025363 |
| MACROPHAGES M1_FANTOM_1 | MACROPHAGES M1_FANTOM_1 | 0.5966326 | 1.6835611 | 0 | 0.00338787 |
| HEPATOCYTES_HPCA_3 | HEPATOCYTES_HPCA_3 | 0.542788 | 1.6683027 | 0 | 0.00420349 |
| HEPATOCYTES_HPCA_1 | HEPATOCYTES_HPCA_1 | 0.53399634 | 1.6602495 | 0 | 0.00466588 |
| CD8+ T-CELLS_IRIS_3 | CD8+ T-CELLS_IRIS_3 | 0.72721213 | 1.644496 | 0.00410959 | 0.00576069 |
| MACROPHAGES_BLUEPRINT_2 | MACROPHAGES_BLUEPRINT_2 | 0.5370811 | 1.6399581 | 0 | 0.00604066 |
| PLASMA CELLS_BLUEPRINT_2 | PLASMA CELLS_BLUEPRINT_2 | 0.52261364 | 1.6119934 | 0 | 0.0089984 |
| CMP_BLUEPRINT_2 | CMP_BLUEPRINT_2 | 0.6717568 | 1.6081461 | 0.02365309 | 0.0095267 |
| MAST CELLS_FANTOM_2 | MAST CELLS_FANTOM_2 | 0.5332538 | 1.5983987 | 0.00210084 | 0.0107194 |
| CD4+ TCM_BLUEPRINT_2 | CD4+ TCM_BLUEPRINT_2 | 0.59867036 | 1.5973246 | 0.00727273 | 0.01083214 |
| CD4+ NAIVE T-CELLS_HPCA_1 | CD4+ NAIVE T-CELLS_HPCA_1 | 0.60289735 | 1.5900215 | 0.01101591 | 0.01185835 |
| CD4+ TCM_BLUEPRINT_3 | CD4+ TCM_BLUEPRINT_3 | 0.53557336 | 1.5785053 | 0.00213904 | 0.01358402 |
| BASOPHILS_FANTOM_1 | BASOPHILS_FANTOM_1 | 0.583328 | 1.5652308 | 0.00960384 | 0.01588941 |
| CMP_BLUEPRINT_3 | CMP_BLUEPRINT_3 | 0.6901126 | 1.5633069 | 0.01641587 | 0.0161354 |
| MESANGIAL CELLS_FANTOM_2 | MESANGIAL CELLS_FANTOM_2 | 0.60990125 | 1.5515505 | 0.01486989 | 0.01856263 |
| TREGS_FANTOM_2 | TREGS_FANTOM_2 | 0.6815786 | 1.5461614 | 0.03076923 | 0.01971826 |
| NEUTROPHILS_IRIS_3 | NEUTROPHILS_IRIS_3 | 0.66361815 | 1.5435333 | 0.02297297 | 0.0201491 |
| MACROPHAGES_FANTOM_1 | MACROPHAGES_FANTOM_1 | 0.65864205 | 1.541494 | 0.0282638 | 0.02054006 |
| MACROPHAGES_IRIS_3 | MACROPHAGES_IRIS_3 | 0.65864205 | 1.5360676 | 0.03924222 | 0.02173273 |
| TREGS_BLUEPRINT_3 | TREGS_BLUEPRINT_3 | 0.6815786 | 1.5342584 | 0.03561644 | 0.02200802 |
| CD4+ TEM_HPCA_1 | CD4+ TEM_HPCA_1 | 0.49547496 | 1.5320772 | 0 | 0.02246572 |
| MACROPHAGES M2_HPCA_2 | MACROPHAGES M2_HPCA_2 | 0.68021667 | 1.5281677 | 0.0269179 | 0.02329962 |
| MAST CELLS_FANTOM_1 | MAST CELLS_FANTOM_1 | 0.4993661 | 1.523387 | 0.00209424 | 0.02436191 |
| HEPATOCYTES_HPCA_2 | HEPATOCYTES_HPCA_2 | 0.4861877 | 1.5233647 | 0 | 0.0242025 |
| CD8+ NAIVE T-CELLS_HPCA_2 | CD8+ NAIVE T-CELLS_HPCA_2 | 0.6119302 | 1.5163641 | 0.02363184 | 0.0259888 |
| PLASMA CELLS_BLUEPRINT_3 | PLASMA CELLS_BLUEPRINT_3 | 0.49112427 | 1.5151616 | 0 | 0.02620952 |
| MESANGIAL CELLS_ENCODE_1 | MESANGIAL CELLS_ENCODE_1 | 0.6703886 | 1.4955252 | 0.02710027 | 0.03240994 |
| MONOCYTES_HPCA_3 | MONOCYTES_HPCA_3 | 0.48408064 | 1.4953116 | 0.00417101 | 0.03230313 |
| MELANOCYTES_ENCODE_1 | MELANOCYTES_ENCODE_1 | 0.6587741 | 1.4939324 | 0.03230337 | 0.03251706 |
| TH1 CELLS_IRIS_1 | TH1 CELLS_IRIS_1 | 0.59663874 | 1.4821361 | 0.03209243 | 0.03651178 |
| CMP_HPCA_1 | CMP_HPCA_1 | 0.472586 | 1.4800729 | 0 | 0.03693071 |
| MACROPHAGES_FANTOM_2 | MACROPHAGES_FANTOM_2 | 0.5633236 | 1.479807 | 0.03194103 | 0.03679765 |
| BASOPHILS_FANTOM_2 | BASOPHILS_FANTOM_2 | 0.4694415 | 1.4542929 | 0.00202224 | 0.04678373 |
| TH2 CELLS_IRIS_3 | TH2 CELLS_IRIS_3 | 0.6411356 | 1.450743 | 0.04471545 | 0.04798172 |
| HSC_BLUEPRINT_3 | HSC_BLUEPRINT_3 | 0.6227081 | 1.4486842 | 0.04699739 | 0.04872888 |
| NEUTROPHILS_IRIS_2 | NEUTROPHILS_IRIS_2 | 0.6529766 | 1.444385 | 0.05146036 | 0.05051168 |
| MONOCYTES_IRIS_1 | MONOCYTES_IRIS_1 | 0.616521 | 1.4389505 | 0.06088083 | 0.05264584 |
| CD4+ NAIVE T-CELLS_HPCA_2 | CD4+ NAIVE T-CELLS_HPCA_2 | 0.5919429 | 1.4205334 | 0.05347594 | 0.06169789 |
| CD4+ NAIVE T-CELLS_HPCA_3 | CD4+ NAIVE T-CELLS_HPCA_3 | 0.5641381 | 1.4192787 | 0.0625 | 0.06192393 |
| TH1 CELLS_IRIS_3 | TH1 CELLS_IRIS_3 | 0.60568213 | 1.4144839 | 0.07310345 | 0.06422797 |
| MONOCYTES_BLUEPRINT_2 | MONOCYTES_BLUEPRINT_2 | 0.59009504 | 1.3927048 | 0.08764941 | 0.07701921 |
| CD8+ NAIVE T-CELLS_HPCA_1 | CD8+ NAIVE T-CELLS_HPCA_1 | 0.48862672 | 1.3925067 | 0.04685715 | 0.07666874 |
| MONOCYTES_HPCA_2 | MONOCYTES_HPCA_2 | 0.61475223 | 1.3842182 | 0.0617284 | 0.08152725 |
| MV ENDOTHELIAL CELLS_FANTOM_1 | MV ENDOTHELIAL CELLS_FANTOM_1 | 0.4538141 | 1.3647335 | 0.03691983 | 0.09505663 |
| MACROPHAGES_FANTOM_3 | MACROPHAGES_FANTOM_3 | 0.48839948 | 1.3617692 | 0.07126168 | 0.0968113 |
| MELANOCYTES_ENCODE_2 | MELANOCYTES_ENCODE_2 | 0.5516381 | 1.3446178 | 0.10741688 | 0.11006428 |
| MACROPHAGES_HPCA_3 | MACROPHAGES_HPCA_3 | 0.44395888 | 1.3378406 | 0.04096639 | 0.11519268 |
| NEURONS_FANTOM_3 | NEURONS_FANTOM_3 | 0.42660314 | 1.3264691 | 0.02886598 | 0.12477575 |
| MACROPHAGES M2_BLUEPRINT_1 | MACROPHAGES M2_BLUEPRINT_1 | 0.4431453 | 1.3108535 | 0.07534984 | 0.13910072 |
| MACROPHAGES_HPCA_2 | MACROPHAGES_HPCA_2 | 0.44745716 | 1.3059723 | 0.08942203 | 0.14346401 |
| MV ENDOTHELIAL CELLS_FANTOM_2 | MV ENDOTHELIAL CELLS_FANTOM_2 | 0.45708603 | 1.2958817 | 0.10402685 | 0.15328929 |
| HSC_BLUEPRINT_2 | HSC_BLUEPRINT_2 | 0.5383281 | 1.2862387 | 0.1521164 | 0.16319937 |
| MACROPHAGES_IRIS_1 | MACROPHAGES_IRIS_1 | 0.48608243 | 1.2789651 | 0.14663461 | 0.17048468 |
| MONOCYTES_IRIS_3 | MONOCYTES_IRIS_3 | 0.48848447 | 1.2782066 | 0.12215569 | 0.17051275 |
| CMP_BLUEPRINT_1 | CMP_BLUEPRINT_1 | 0.54485244 | 1.2768072 | 0.18134034 | 0.17094477 |
| PREADIPOCYTES_FANTOM_1 | PREADIPOCYTES_FANTOM_1 | 0.51829016 | 1.255251 | 0.19205298 | 0.19593377 |
| MACROPHAGES_HPCA_1 | MACROPHAGES_HPCA_1 | 0.418549 | 1.2544471 | 0.08709339 | 0.19599564 |
| MESANGIAL CELLS_ENCODE_3 | MESANGIAL CELLS_ENCODE_3 | 0.43369547 | 1.2495638 | 0.11891279 | 0.2012018 |
| NEURONS_FANTOM_1 | NEURONS_FANTOM_1 | 0.4081767 | 1.2300544 | 0.13185655 | 0.2260329 |
| MACROPHAGES_IRIS_2 | MACROPHAGES_IRIS_2 | 0.4153799 | 1.2139096 | 0.16079295 | 0.24804796 |
| NEURONS_FANTOM_2 | NEURONS_FANTOM_2 | 0.38661674 | 1.2107506 | 0.09713701 | 0.25148857 |
| CD4+ TCM_BLUEPRINT_1 | CD4+ TCM_BLUEPRINT_1 | 0.4437387 | 1.2072977 | 0.19927536 | 0.25523567 |
| MONOCYTES_HPCA_1 | MONOCYTES_HPCA_1 | 0.50392455 | 1.1954992 | 0.23476005 | 0.27213067 |
| PLATELETS_HPCA_1 | PLATELETS_HPCA_1 | 0.38419455 | 1.1831195 | 0.16892597 | 0.2905218 |
| CD4+ MEMORY T-CELLS_FANTOM_3 | CD4+ MEMORY T-CELLS_FANTOM_3 | 0.3808735 | 1.1686577 | 0.19815195 | 0.31309572 |
| PRO B-CELLS_HPCA_2 | PRO B-CELLS_HPCA_2 | 0.3753764 | 1.1663754 | 0.18640576 | 0.31520587 |
| MESANGIAL CELLS_FANTOM_1 | MESANGIAL CELLS_FANTOM_1 | 0.3860989 | 1.1448622 | 0.23541887 | 0.3511447 |
| CD4+ MEMORY T-CELLS_FANTOM_1 | CD4+ MEMORY T-CELLS_FANTOM_1 | 0.35827577 | 1.1223073 | 0.22920893 | 0.38964722 |
| MEP_HPCA_1 | MEP_HPCA_1 | 0.45121664 | 1.1080313 | 0.35923567 | 0.4146093 |
| FIBROBLASTS_HPCA_1 | FIBROBLASTS_HPCA_1 | 0.43003994 | 1.1038122 | 0.3458283 | 0.4204343 |
| PRO B-CELLS_HPCA_1 | PRO B-CELLS_HPCA_1 | 0.3903916 | 1.0899075 | 0.3631157 | 0.44507417 |
| MEP_HPCA_2 | MEP_HPCA_2 | 0.43071204 | 1.0877583 | 0.35872236 | 0.44679174 |
| ENDOTHELIAL CELLS_FANTOM_3 | ENDOTHELIAL CELLS_FANTOM_3 | 0.40115875 | 1.082552 | 0.37032712 | 0.45515734 |
| MACROPHAGES M2_BLUEPRINT_3 | MACROPHAGES M2_BLUEPRINT_3 | 0.40869248 | 1.0820992 | 0.35961768 | 0.45373696 |
| MEP_NOVERSHTERN_3 | MEP_NOVERSHTERN_3 | 0.43071204 | 1.0792714 | 0.36684072 | 0.4567452 |
| ERYTHROCYTES_FANTOM_2 | ERYTHROCYTES_FANTOM_2 | 0.46949047 | 1.0753769 | 0.38283378 | 0.46171185 |
| MESANGIAL CELLS_FANTOM_3 | MESANGIAL CELLS_FANTOM_3 | 0.34026083 | 1.0613195 | 0.36485097 | 0.48667988 |
| PLATELETS_HPCA_2 | PLATELETS_HPCA_2 | 0.3414356 | 1.0536131 | 0.38848922 | 0.4998397 |
| PREADIPOCYTES_FANTOM_3 | PREADIPOCYTES_FANTOM_3 | 0.39837462 | 1.0361501 | 0.43309003 | 0.53211653 |
| MYOCYTES_ENCODE_2 | MYOCYTES_ENCODE_2 | 0.43839413 | 1.0149237 | 0.46341464 | 0.57217807 |
| ADIPOCYTES_HPCA_3 | ADIPOCYTES_HPCA_3 | 0.44673085 | 1.0141714 | 0.47599453 | 0.5707755 |
| LY ENDOTHELIAL CELLS_FANTOM_2 | LY ENDOTHELIAL CELLS_FANTOM_2 | 0.37187216 | 1.0069803 | 0.47585395 | 0.58250755 |
| CMP_NOVERSHTERN_3 | CMP_NOVERSHTERN_3 | 0.4193415 | 1.0006274 | 0.47814208 | 0.5920601 |
| FIBROBLASTS_FANTOM_3 | FIBROBLASTS_FANTOM_3 | 0.3654692 | 0.98781604 | 0.50473934 | 0.6148299 |
| NEUTROPHILS_FANTOM_2 | NEUTROPHILS_FANTOM_2 | 0.34370765 | 0.97824466 | 0.5355932 | 0.63022095 |
| HSC_FANTOM_1 | HSC_FANTOM_1 | 0.4409872 | 0.9716764 | 0.53203344 | 0.64010805 |
| PRO B-CELLS_NOVERSHTERN_1 | PRO B-CELLS_NOVERSHTERN_1 | 0.33600038 | 0.95079726 | 0.56766057 | 0.6782163 |
| SMOOTH MUSCLE_ENCODE_3 | SMOOTH MUSCLE_ENCODE_3 | 0.33201027 | 0.93596363 | 0.59246576 | 0.70369047 |
| MACROPHAGES M2_HPCA_1 | MACROPHAGES M2_HPCA_1 | 0.31745195 | 0.9287667 | 0.6035242 | 0.714145 |
| GMP_BLUEPRINT_3 | GMP_BLUEPRINT_3 | 0.3208023 | 0.9207283 | 0.59639233 | 0.72597206 |
| FIBROBLASTS_HPCA_2 | FIBROBLASTS_HPCA_2 | 0.31866425 | 0.9148784 | 0.63656884 | 0.7334162 |
| EPITHELIAL CELLS_FANTOM_1 | EPITHELIAL CELLS_FANTOM_1 | 0.3984222 | 0.9105873 | 0.60422164 | 0.7377012 |
| NEURONS_ENCODE_3 | NEURONS_ENCODE_3 | 0.30099717 | 0.89006406 | 0.69239014 | 0.770811 |
| KERATINOCYTES_HPCA_1 | KERATINOCYTES_HPCA_1 | 0.3767261 | 0.8762278 | 0.66319895 | 0.7913624 |
| GMP_BLUEPRINT_1 | GMP_BLUEPRINT_1 | 0.30273965 | 0.876045 | 0.69349504 | 0.7879914 |
| PRO B-CELLS_HPCA_3 | PRO B-CELLS_HPCA_3 | 0.30565456 | 0.8434516 | 0.7071759 | 0.8388055 |
| ENDOTHELIAL CELLS_ENCODE_2 | ENDOTHELIAL CELLS_ENCODE_2 | 0.3036816 | 0.8266104 | 0.7322275 | 0.8612211 |
| ERYTHROCYTES_NOVERSHTERN_3 | ERYTHROCYTES_NOVERSHTERN_3 | 0.2732656 | 0.81297904 | 0.8142251 | 0.8777771 |
| ENDOTHELIAL CELLS_HPCA_3 | ENDOTHELIAL CELLS_HPCA_3 | 0.28002763 | 0.806006 | 0.80893856 | 0.88416314 |
| MV ENDOTHELIAL CELLS_HPCA_1 | MV ENDOTHELIAL CELLS_HPCA_1 | 0.345036 | 0.7958731 | 0.7533693 | 0.8945589 |
| FIBROBLASTS_FANTOM_1 | FIBROBLASTS_FANTOM_1 | 0.25591296 | 0.7783382 | 0.8772112 | 0.9149078 |
| FIBROBLASTS_HPCA_3 | FIBROBLASTS_HPCA_3 | 0.25591296 | 0.77492845 | 0.8759124 | 0.9154718 |
| SKELETAL MUSCLE_FANTOM_1 | SKELETAL MUSCLE_FANTOM_1 | 0.25841346 | 0.7670465 | 0.86147183 | 0.921292 |
| SKELETAL MUSCLE_FANTOM_2 | SKELETAL MUSCLE_FANTOM_2 | 0.26279837 | 0.76105994 | 0.8566703 | 0.924769 |
| SKELETAL MUSCLE_FANTOM_3 | SKELETAL MUSCLE_FANTOM_3 | 0.26190618 | 0.7574938 | 0.86263734 | 0.92535174 |
| ENDOTHELIAL CELLS_ENCODE_3 | ENDOTHELIAL CELLS_ENCODE_3 | 0.29746976 | 0.74664295 | 0.8053777 | 0.93473655 |
| EPITHELIAL CELLS_HPCA_3 | EPITHELIAL CELLS_HPCA_3 | 0.31182536 | 0.73839617 | 0.8144611 | 0.940252 |
| GMP_BLUEPRINT_2 | GMP_BLUEPRINT_2 | 0.24374972 | 0.7228516 | 0.91550803 | 0.95344967 |
| ENDOTHELIAL CELLS_ENCODE_1 | ENDOTHELIAL CELLS_ENCODE_1 | 0.25731358 | 0.7106961 | 0.88119954 | 0.9622436 |
| MYOCYTES_FANTOM_1 | MYOCYTES_FANTOM_1 | 0.31752777 | 0.7040172 | 0.8491848 | 0.964565 |
| ENDOTHELIAL CELLS_BLUEPRINT_3 | ENDOTHELIAL CELLS_BLUEPRINT_3 | 0.22177674 | 0.6804561 | 0.96149844 | 0.9819808 |
| PERICYTES_ENCODE_1 | PERICYTES_ENCODE_1 | 0.26770994 | 0.66283387 | 0.8992347 | 0.99222994 |
| CMP_HPCA_2 | CMP_HPCA_2 | 0.22678292 | 0.65888524 | 0.95788336 | 0.9909469 |
| NEUTROPHILS_FANTOM_3 | NEUTROPHILS_FANTOM_3 | 0.25008282 | 0.65802723 | 0.9085511 | 0.9873163 |
| CLP_BLUEPRINT_3 | CLP_BLUEPRINT_3 | 0.24882562 | 0.63870764 | 0.9288344 | 0.9960951 |
| MV ENDOTHELIAL CELLS_ENCODE_3 | MV ENDOTHELIAL CELLS_ENCODE_3 | 0.20499818 | 0.633697 | 0.98956156 | 0.9950837 |
| CLP_BLUEPRINT_2 | CLP_BLUEPRINT_2 | 0.2818171 | 0.63116175 | 0.92027026 | 0.99263483 |
| NEUTROPHILS_FANTOM_1 | NEUTROPHILS_FANTOM_1 | 0.22604966 | 0.6121043 | 0.96378505 | 0.99876636 |
| MV ENDOTHELIAL CELLS_HPCA_3 | MV ENDOTHELIAL CELLS_HPCA_3 | 0.25422505 | 0.6086501 | 0.92724866 | 0.9962571 |
| GMP_HPCA_1 | GMP_HPCA_1 | 0.21077466 | 0.6084512 | 0.98476607 | 0.99222463 |
| SMOOTH MUSCLE_ENCODE_2 | SMOOTH MUSCLE_ENCODE_2 | 0.22475596 | 0.5719118 | 0.955243 | 1 |
| MV ENDOTHELIAL CELLS_ENCODE_1 | MV ENDOTHELIAL CELLS_ENCODE_1 | 0.18150638 | 0.56415147 | 0.99693567 | 1 |
| SMOOTH MUSCLE_FANTOM_2 | SMOOTH MUSCLE_FANTOM_2 | 0.22475596 | 0.56133765 | 0.9680307 | 0.9970731 |
| MV ENDOTHELIAL CELLS_ENCODE_2 | MV ENDOTHELIAL CELLS_ENCODE_2 | 0.173715 | 0.5402462 | 0.9989744 | 0.998469 |
| MPP_BLUEPRINT_1 | MPP_BLUEPRINT_1 | 0.1871273 | 0.5125381 | 0.995338 | 0.9996221 |
| MPP_BLUEPRINT_2 | MPP_BLUEPRINT_2 | 0.17326578 | 0.50955254 | 0.9967533 | 0.99609137 |
| SMOOTH MUSCLE_HPCA_3 | SMOOTH MUSCLE_HPCA_3 | 0.15727167 | 0.4787544 | 1 | 0.99547493 |
| **Cell Signature vs LN Neg - Downregulated genes** | |  |  |  |  |
| **NAME** | **GS<br> follow link to MSigDB** | **ES** | **NES** | **NOM p-val** | **FDR q-val** |
| SEBOCYTES_FANTOM_1 | SEBOCYTES_FANTOM_1 | -0.6022862 | -2.0213144 | 0 | 0.02403197 |
| KERATINOCYTES_FANTOM_3 | KERATINOCYTES_FANTOM_3 | -0.5127857 | -2.0206325 | 0 | 0.01201598 |
| KERATINOCYTES_FANTOM_2 | KERATINOCYTES_FANTOM_2 | -0.5087371 | -1.9142565 | 0 | 0.02486576 |
| SEBOCYTES_FANTOM_2 | SEBOCYTES_FANTOM_2 | -0.6167682 | -1.9005115 | 0 | 0.02082684 |
| SEBOCYTES_FANTOM_3 | SEBOCYTES_FANTOM_3 | -0.5175234 | -1.8157078 | 0 | 0.04082391 |
| KERATINOCYTES_FANTOM_1 | KERATINOCYTES_FANTOM_1 | -0.4018575 | -1.6772386 | 0 | 0.1038389 |
| KERATINOCYTES_ENCODE_2 | KERATINOCYTES_ENCODE_2 | -0.5670557 | -1.6371359 | 0.0125 | 0.11838067 |
| ASTROCYTES_FANTOM_2 | ASTROCYTES_FANTOM_2 | -0.3961215 | -1.6263267 | 0.01333333 | 0.11270441 |
| CHONDROCYTES_ENCODE_3 | CHONDROCYTES_ENCODE_3 | -0.4418136 | -1.6180618 | 0.00662252 | 0.10638983 |
| CHONDROCYTES_FANTOM_2 | CHONDROCYTES_FANTOM_2 | -0.3805357 | -1.6105719 | 0 | 0.10160988 |
| KERATINOCYTES_HPCA_3 | KERATINOCYTES_HPCA_3 | -0.4317913 | -1.562021 | 0.00613497 | 0.12550876 |
| CHONDROCYTES_HPCA_1 | CHONDROCYTES_HPCA_1 | -0.5384843 | -1.4988981 | 0.05714286 | 0.17200251 |
| CHONDROCYTES_ENCODE_1 | CHONDROCYTES_ENCODE_1 | -0.4841684 | -1.4820032 | 0.048583 | 0.17575817 |
| ASTROCYTES_FANTOM_3 | ASTROCYTES_FANTOM_3 | -0.3606343 | -1.4810312 | 0 | 0.1646015 |
| ASTROCYTES_FANTOM_1 | ASTROCYTES_FANTOM_1 | -0.3210259 | -1.472934 | 0 | 0.16234784 |
| MSC_FANTOM_3 | MSC_FANTOM_3 | -0.5099998 | -1.4668736 | 0.05639098 | 0.15667069 |
| MSC_HPCA_1 | MSC_HPCA_1 | -0.4149413 | -1.4475375 | 0.04142012 | 0.16801502 |
| CHONDROCYTES_ENCODE_2 | CHONDROCYTES_ENCODE_2 | -0.4625195 | -1.4355025 | 0.06481481 | 0.16802475 |
| EPITHELIAL CELLS_ENCODE_3 | EPITHELIAL CELLS_ENCODE_3 | -0.3338514 | -1.4093652 | 0.0326087 | 0.18424565 |
| CHONDROCYTES_FANTOM_3 | CHONDROCYTES_FANTOM_3 | -0.3210149 | -1.3950614 | 0 | 0.18953417 |
| OSTEOBLAST_FANTOM_3 | OSTEOBLAST_FANTOM_3 | -0.3391475 | -1.3507782 | 0.04310345 | 0.23249464 |
| FIBROBLASTS_ENCODE_3 | FIBROBLASTS_ENCODE_3 | -0.4043629 | -1.3331949 | 0.10112359 | 0.24421701 |
| MYOCYTES_FANTOM_2 | MYOCYTES_FANTOM_2 | -0.3993017 | -1.3311507 | 0.10869565 | 0.2357215 |
| PREADIPOCYTES_ENCODE_1 | PREADIPOCYTES_ENCODE_1 | -0.4129774 | -1.3243839 | 0.1 | 0.23397301 |
| MSC_HPCA_2 | MSC_HPCA_2 | -0.4522835 | -1.3213447 | 0.11417323 | 0.2278325 |
| ASTROCYTES_ENCODE_1 | ASTROCYTES_ENCODE_1 | -0.3561043 | -1.3195097 | 0.06015038 | 0.22111875 |
| CHONDROCYTES_FANTOM_1 | CHONDROCYTES_FANTOM_1 | -0.2893929 | -1.2982299 | 0 | 0.23907529 |
| FIBROBLASTS_ENCODE_2 | FIBROBLASTS_ENCODE_2 | -0.3116466 | -1.2629197 | 0.08421053 | 0.27925536 |
| ASTROCYTES_ENCODE_3 | ASTROCYTES_ENCODE_3 | -0.436449 | -1.2528628 | 0.20083682 | 0.28410268 |
| KERATINOCYTES_HPCA_2 | KERATINOCYTES_HPCA_2 | -0.3268505 | -1.2510868 | 0.1119403 | 0.27689123 |
| MEP_NOVERSHTERN_1 | MEP_NOVERSHTERN_1 | -0.3304807 | -1.2457746 | 0.12173913 | 0.2754961 |
| NEURONS_ENCODE_2 | NEURONS_ENCODE_2 | -0.2718376 | -1.2092682 | 0.03333334 | 0.31766048 |
| OSTEOBLAST_FANTOM_1 | OSTEOBLAST_FANTOM_1 | -0.3423687 | -1.2006795 | 0.16666667 | 0.32211146 |
| GMP_HPCA_3 | GMP_HPCA_3 | -0.271947 | -1.1917208 | 0.09090909 | 0.3262726 |
| KERATINOCYTES_ENCODE_1 | KERATINOCYTES_ENCODE_1 | -0.3692899 | -1.180262 | 0.21105528 | 0.33433393 |
| FIBROBLASTS_FANTOM_2 | FIBROBLASTS_FANTOM_2 | -0.3946306 | -1.1332473 | 0.28957528 | 0.40453357 |
| EPITHELIAL CELLS_FANTOM_2 | EPITHELIAL CELLS_FANTOM_2 | -0.3131318 | -1.1196901 | 0.2635135 | 0.4180748 |
| SMOOTH MUSCLE_FANTOM_3 | SMOOTH MUSCLE_FANTOM_3 | -0.2911362 | -1.1169568 | 0.21818182 | 0.41252467 |
| ASTROCYTES_ENCODE_2 | ASTROCYTES_ENCODE_2 | -0.2961289 | -1.0943449 | 0.23880596 | 0.44461036 |
| OSTEOBLAST_FANTOM_2 | OSTEOBLAST_FANTOM_2 | -0.3131535 | -1.090671 | 0.3243243 | 0.44062302 |
| FIBROBLASTS_ENCODE_1 | FIBROBLASTS_ENCODE_1 | -0.2202602 | -0.958168 | 0.5714286 | 0.74275094 |
| MV ENDOTHELIAL CELLS_HPCA_2 | MV ENDOTHELIAL CELLS_HPCA_2 | -0.2877252 | -0.9390672 | 0.57608694 | 0.7790874 |
| EPITHELIAL CELLS_FANTOM_3 | EPITHELIAL CELLS_FANTOM_3 | -0.3020258 | -0.9382239 | 0.5458937 | 0.763406 |
| CMP_NOVERSHTERN_1 | CMP_NOVERSHTERN_1 | -0.2809772 | -0.9166103 | 0.5706806 | 0.8024189 |
| EPITHELIAL CELLS_ENCODE_2 | EPITHELIAL CELLS_ENCODE_2 | -0.290145 | -0.9071313 | 0.6064257 | 0.8061099 |
| MSC_HPCA_3 | MSC_HPCA_3 | -0.2272406 | -0.9048457 | 0.775 | 0.79366547 |
| MPP_BLUEPRINT_3 | MPP_BLUEPRINT_3 | -0.2412814 | -0.8973365 | 0.6721311 | 0.79360825 |
| MV ENDOTHELIAL CELLS_FANTOM_3 | MV ENDOTHELIAL CELLS_FANTOM_3 | -0.1998241 | -0.8505927 | 0.86 | 0.88027775 |
| ERYTHROCYTES_NOVERSHTERN_1 | ERYTHROCYTES_NOVERSHTERN_1 | -0.2093634 | -0.8465037 | 0.84090906 | 0.8704295 |
| PERICYTES_ENCODE_3 | PERICYTES_ENCODE_3 | -0.1893971 | -0.8150278 | 0.90909094 | 0.91087294 |
| OSTEOBLAST_HPCA_3 | OSTEOBLAST_HPCA_3 | -0.2048995 | -0.8089786 | 0.9230769 | 0.90337574 |
| ERYTHROCYTES_NOVERSHTERN_2 | ERYTHROCYTES_NOVERSHTERN_2 | -0.2248725 | -0.7845081 | 0.85714287 | 0.92526 |
| MYOCYTES_ENCODE_3 | MYOCYTES_ENCODE_3 | -0.1674366 | -0.7663041 | 1 | 0.93303055 |
| SMOOTH MUSCLE_FANTOM_1 | SMOOTH MUSCLE_FANTOM_1 | -0.1640376 | -0.7475588 | 1 | 0.93947446 |
| OSTEOBLAST_HPCA_1 | OSTEOBLAST_HPCA_1 | -0.2046356 | -0.7276933 | 0.9378531 | 0.94349277 |
| PREADIPOCYTES_ENCODE_3 | PREADIPOCYTES_ENCODE_3 | -0.1536309 | -0.6646855 | 1 | 0.9777879 |
| OSTEOBLAST_HPCA_2 | OSTEOBLAST_HPCA_2 | -0.2369559 | -0.6554093 | 0.89873415 | 0.9658758 |
| PERICYTES_ENCODE_2 | PERICYTES_ENCODE_2 | -0.1428532 | -0.6102946 | 1 | 0.9706012 |

**Supplementary Table 8.**Demographics of patients in the validation set (AMC-AJCCll-90).

| Characteristics | 90 patients Stage ll |
| --- | --- |
| Male sex (%) | 42(46.7%) |
| Median at age at the time of operation  (range) | 73.4(34.6-95.1) |
| T-stage  -T3  -T4 | 81(90%)  9(10%) |
| Location  -Left  -Right | 38(42.2%)  52(57.8%) |
| Lymph node yield  (range) | 12(1-46) |

**Supplementary Table 9.**GSEA data of hallmark gene sets enriched or de-enriched in AMC-AJCCll-90.

**Supplementary Figure 1.** (A-C) GSEA plots of the most enriched hallmark gene sets in low LN yield colon tumours.


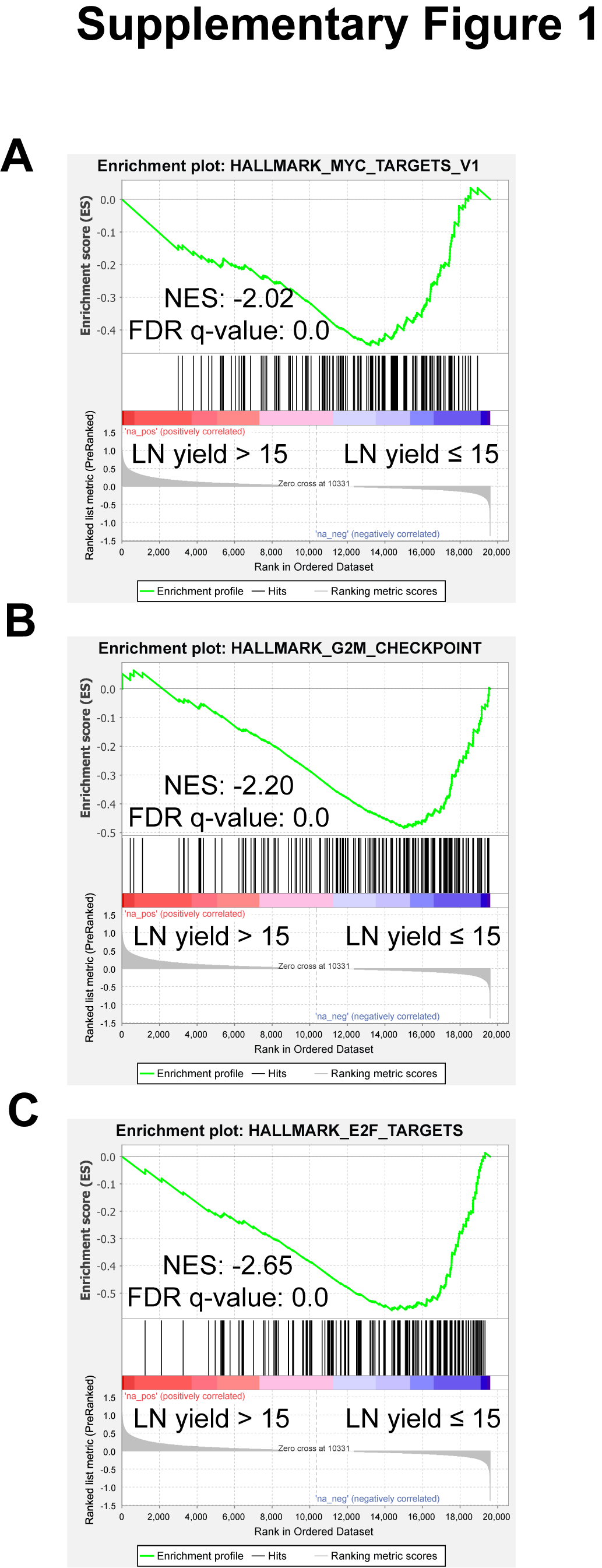

Supplement: Supplementary file 2 — Supplementary Table and Figures [file 41416_2022_1700_MOESM2_ESM.docx]
